# Development and validation of the Vaccine Barriers Assessment Tool for identifying drivers of under-vaccination in children under five years in Australia

**DOI:** 10.1080/21645515.2024.2359623

**Published:** 2024-06-06

**Authors:** Jessica Kaufman, Jane Tuckerman, Carissa Bonner, David N. Durrheim, Daniel S. J.3 Costa, Lyndal Trevena, Jörg Henseler, Margie Danchin

**Affiliations:** a Vaccine Uptake Group, Murdoch Children’s Research Institute, Royal Children’s Hospital, Parkville, Australia; b Department of Paediatrics, The University of Melbourne, Royal Children’s Hospital, Parkville, Australia; c School of Public Health, University of Sydney, Camperdown, Australia; d School of Medicine and Public Health, University of Newcastle, University Drive, Callaghan, Australia; e Health Protection, Hunter New England Population Health, Wallsend, Australia; f School of Psychology, University of Sydney, Camperdown, Australia; g Department of Design, Production & Management, University of Twente, Enschede, Netherlands; h Nova Information Management School, Universidade Nova de Lisboa, Lisbon, Portugal; i Department of General Medicine, Royal Children’s Hospital, Parkville, Australia

**Keywords:** Childhood vaccination, immunization, barriers, parents, children, vaccine acceptance, knowledge, attitude, access, measurement, scale

## Abstract

Data on routine childhood vaccination coverage can only tell us who is under-vaccinated; it cannot explain why vaccine coverage is low. Collecting data on the reasons behind under-vaccination is necessary to implement cost-effective strategies that address key barriers and target interventions appropriately. However, no instruments that measure both vaccine acceptance and access factors among parents of children <5 y have been validated in high-income countries. This study aims to develop and validate the Vaccine Barriers Assessment Tool (VBAT) for Australia. We applied three phases of mixed methods data collection and analysis. In Phase 1, we developed a comprehensive list of 80 items reflecting all potential parental barriers to childhood vaccination, derived from published literature and behavioral theory. Through cognitive interviews (*n* = 28), we refined this list to 45 items. In Phase 2, we conducted a two-wave online survey to test the reliability and validity of these items in an Australian sample of parents (*n* = 532) with structural equation modeling, further refining the list to 35 items. In Phase 3, we conducted a final parent survey (*n* = 156), administering these items along with the Parent Attitudes toward Childhood Vaccination (PACV) scale for comparison. We reviewed participants’ immunization register data to assess the predictive validity of the proposed models. The final 6-item short form and 15-item long form Vaccine Barriers Assessment Tool assess access, communal benefit, personal risk, equity, commitment, social norms, and trust in health-care workers. It is being applied for national surveillance in Australia and will be adapted for additional populations and vaccines.

## Introduction

As the world begins to recover from the global health emergency of COVID-19, assessing barriers to routine childhood vaccination to improve vaccine uptake and parental vaccine confidence has emerged as an urgent priority.^1^ Globally, between 2019 and 2021, more than 67 million children under age 5 missed one or more doses of recommended routine vaccines, and measles vaccination coverage declined from 86% to 81%, the largest sustained backslide in immunization in 30 y.^1^ The largest drops in childhood immunization coverage have been experienced in low- and middle-income countries.^1^ However, declines in coverage have also been recorded in high-income countries. For example, in the United Kingdom, measles vaccine coverage by age five is at its lowest rate in 10 y (85.2%),^2^ resulting in significant surges in measles cases in 2023 and 2024.^3^ New Zealand vaccine coverage among 2-y-olds is 82.5%, a drop of nearly 8% since 2020.^4^ In Australia, vaccine coverage has dropped below the 95% target for all age groups, with 2-y-old coverage now at 91.2% in the general population and 88.8% among Aboriginal and Torres Strait Islander children.^5,6^

Unlike many other countries, Australia is able to track changes in childhood vaccination rates through comprehensive registers like the Australian Immunisation Register (AIR), which collects basic demographic data to more accurately define specific low coverage populations. However, vaccination rates from the national register do not provide insights into why coverage gaps exist. They cannot identify parents or carers experiencing access challenges nor those with significant concerns who continue to vaccinate but are at risk of non-vaccination in the future. Understanding the changing social and behavioral drivers of vaccination is key to developing targeted, cost-effective vaccination strategies to improve and sustain uptake.

The two main causes of under- or non-vaccination are acceptance or access barriers or a combination of both.^7^ Addressing these barriers requires different strategies and policies. Since the start of the COVID-19 pandemic, there is evidence that parental concerns about vaccination have increased^8,9^ and the belief that vaccines are important for children has decreased in Australia.^1^ To address these and other acceptance-related barriers, tailored, dialogue-based communication interventions are among the most effective strategies.^10^ However, where accessibility issues like getting time off work or traveling to a clinic are the primary barriers, interventions like outreach and extended clinic hours will be more effective than even the most evidence-based communication interventions. Whilst systemic economic and political barriers also impact vaccine uptake, identification of parent-level barriers that can be measured and feasibly addressed is of most value to governments and communities. Importantly, these barriers can change over time, as people are influenced by individual and social factors including exposure to misinformation, disease outbreaks, new vaccine introduction and policy changes.

The COVID-19 pandemic highlighted the need for a valid and repeatable measure of access and acceptance barriers to inform policy decisions and target interventions, but currently there is no systematic way to identify and track the drivers of childhood under-vaccination in Australia. Instruments to measure vaccine acceptance or confidence have been developed and validated for adolescents, children, and certain vaccines, but none of these include assessment of access barriers.^11–19^ Recently, the World Health Organization (WHO) Behavioural and Social Drivers of Vaccination working group developed a flexible set of quantitative and qualitative instruments to help countries measure acceptance as well as access issues, mostly targeted to low- and middle-income country settings.^20,21^ However, these instruments have not been validated against behavior or vaccine uptake in Australia.

Therefore, we aimed to develop and validate both a short and a long form of the Vaccine Barriers Assessment Tool (VBAT) to comprehensively measure drivers of both parental acceptance and access to vaccination in children under 5 y in Australia. The short form can be applied efficiently or combined with other population assessment tools, while the longer form can be used to provide more detailed insights.

## Materials and methods

**Figure 1.**
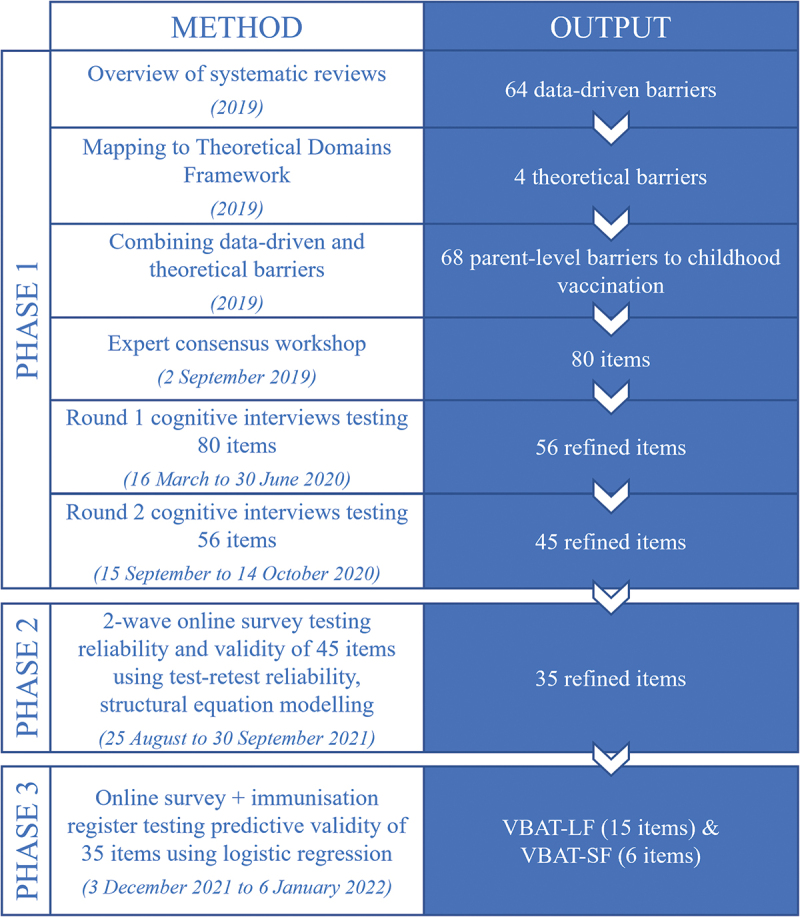
Overview of methods and outputs of three phases of VBAT development and validation.

This manuscript presents the three phases of instrument development and validation, which took place between 2019 and 2023. Beginning with a broad item set derived from both behavioral theory and published research evidence, our goal was to refine and reduce the items to reach a final long (VBAT-LF) and short form (VBAT-SF) of the instrument. In all phases, insights generated through relevant qualitative or quantitative methods were considered and contextualized by the core investigator team, comprising eight experts in vaccinology, pediatric medicine, psychology, psychometrics, and social and behavioral science. The project advisory group, which included public health policymakers and researchers, also provided guidance. Figure 1 shows an overview of the methods and outputs of each phase (explained further in this section). Most aspects of the development and validation process took place primarily in Australia, with some data collection in New Zealand. Additional development and validation in the New Zealand context is underway.

The phases of this project received ethical approval from the Royal Children’s Hospital Human Research Ethics Committee (#60277, February 2020, and #75945, July 2021) and the University of Auckland Human Participants Ethics Committee (#024299, May 2020). Participation in all phases of the study was voluntary, and participants could withdraw until the conclusion of the data collection period. Parents or carers (hereafter referred to as ‘parents’) taking part in Phase 1 cognitive interviews were reimbursed for their time. Parents who completed the Phase 2 and 3 surveys were offered the option of entering a prize draw or earning points to be redeemed for gift vouchers.

### Phase one: development of the initial item set

We aimed to develop a comprehensive initial item set that reflected all potential parental barriers to childhood vaccination. First, we conducted a global overview of qualitative and quantitative systematic reviews of parent-level barriers (i.e., barriers experienced by or relevant to parents) to vaccination of children aged ≤5 y in both high-income and low- and middle-income settings.^7^ We extracted data on all described barriers and inductively grouped them into categories. We then deductively mapped the barriers from the systematic reviews against the categories of the Theoretical Domains Framework. This allowed us to identify additional theoretical barriers not described in the literature, such as barriers related to goal setting (e.g., low prioritization of vaccination).^22^

To draft items for each barrier, we held a 1-d consensus workshop (2 September 2019) with the core investigator team in Australia. Where existing measurement instruments assessed similar barriers (e.g. acceptance tools such as the Parent Attitudes about Childhood Vaccination (PACV),^13^ the Vaccine Hesitancy Scale,^15^ Vaccine Confidence Scale),^17^ we reviewed the wording used in these instruments and aimed to avoid exact duplication. Individual items from a validated scale do not remain ‘validated’ when taken on their own, so there was no advantage to retaining their exact wording. Furthermore, we wanted to ensure that we were guided by the cognitive interviews rather than preconceived bias when selecting or rephrasing items. We framed items positively where possible and followed general criteria for item phrasing, such as avoiding jargon and double-barreled questions. We drafted multiple items for some barriers, particularly those not commonly measured by existing instruments, and included a comprehensive set of access barriers.

We then tested the initial item set in two rounds of cognitive interviews with parents to refine the wording of the questions and response options and ensure face validity.^23^ In round 1 (16 March to 30 June 2020), we tested the full item set (*N* = 80 items) with Australian parents of children under 5 years. Parents were recruited through the Social Research Centre Melbourne by paid social media advertising and expression of interest invitation emailed to the Social Research Centre’s 170 Victorian database members. We used a maximum variation sampling approach to include parents with a first language other than English and with different levels of education, income, and degree of vaccine hesitancy. Vaccine hesitancy was assessed with a single item based on the Vaccine Communication Framework (VCF).^24,25^ The VCF has five levels which we collapsed into three categories: parents who give their child all recommended vaccines and have no vaccine concerns (no hesitancy; VCF level 1), those who vaccinate with minor vaccine concerns (low hesitancy; VCF level 2), and parents who refuse, delay, or vaccinate but have a lot of concerns (high hesitancy; VCF levels 3–5). All interviews were conducted either in person or via Zoom and parents provided written informed consent. Parents were asked to read items aloud and describe their understanding of the item and how they would approach responding to it. We summarized feedback on each item and revised wording or removed from the item set those items with significant comprehension or interpretation issues.

The revised item set (*N* = 56 items) was taken to a second round of cognitive interviews (15 September to 14 October 2020), including interviews conducted in New Zealand with Māori and Pacific parents, to ensure cross-country appropriateness in support of future New Zealand instrument development and validation. New Zealand parents were recruited through social media, e-mail lists, word-of-mouth and the New Zealand team’s networks. Additional parents in Australia at high risk of under-vaccination due to socioeconomic status or hesitancy were recruited via the research team’s networks in the North Coast of NSW and in Newcastle, NSW. Following this second round of cognitive interviewing, the items were revised again to form the VBAT draft 1-item set (*N* = 45 items).

### Phase two: reliability and validity of the VBAT items

We assessed the reliability and validity of the draft 1 item set in an online survey (25 August to 13 September 2021) with parents who were members of an online Australia-wide panel. A sample size of *n* = 450 was determined using the traditional principal component analysis guideline of 10 participants per item,^26^ which we increased to *n* = 500 to ensure we were able to recruit sufficient parents across sampling categories. To avoid non-independence, only one parent from a couple was recruited. Parents were recruited by the Social Research Centre via e-mail invitation using a criterion sampling approach. Parents who were >18 y old with a youngest child aged <5 y were eligible.

The primary sampling criterion was vaccination coverage of residential area, determined by matching postcodes with Australian Bureau of Statistics regional fully immunized coverage data.^5^ We defined three levels based on Australian coverage, which is typically between 90% and 95%,^5^ with anything below that considered “low” and anything above considered “high.” Specifically, low coverage was defined as <90% in either of the reported age brackets (12-<15 month or 24-<27 months). Medium coverage was defined as 90 to <95% and high coverage >95%. We aimed to recruit 40% of participants from low, 20% from medium and 40% from high coverage areas. Additional sampling criteria included level of vaccine hesitancy, determined by the VCF survey question used in Phase 1, and socioeconomic status of the participant’s residential area. Socioeconomic status was determined using postcode and was based on the tertiles of the Australian Bureau of Statistics Socio-Economic Indexes for Areas (SEIFA) Disadvantage Index.^27^ Based on previous community research by our group,^25^ we aimed for our sample to include at least 15% highly vaccine hesitant parents and 40% from both low and high SEIFA tertiles, but these were targets rather than quotas.

Eligible parents completed all 45 draft items and provided demographic details. Consent was implied by survey completion. Parents were invited to complete a re-test of the same items 2 weeks after the first survey (8 to 30 September 2021).

To determine which items should be taken through to phase 3 validation, we calculated the correlation between individual items and the VCF vaccine hesitancy item (not part of the VBAT item set) and between each item’s test and retest response. For each item, we also described the frequency of each response option. The core investigator team discussed and considered the inclusion of any items with potentially poor performance based on the following data: a) construct validity of acceptance-related items, defined as correlation <0.2 with the VCF vaccine hesitancy item; b) test–retest reliability, defined by the intercorrelation coefficient <0.3; c) ceiling effect, which we defined as >70% responding “strongly agree;” and d) >5% responding “don’t know/can’t say.” All these data were considered by the investigator team, along with and qualitative factors like relevance to policymaking and equity, with the final decision about which items to include in the VBAT draft 2 item set based on the expert consensus.

### Phase three: predictive validity of VBAT model

We assessed the predictive validity of the VBAT draft 2 items (*N* = 35) in a second survey (3 December 2021 to 6 January 2022) using a convenience sample of different parents recruited from the same online panel as used in phase two. Based on the number of items progressed for validation testing in this phase, we aimed to recruit *n* = 350 parents. As we needed to assess behavior or future vaccine uptake in this validation phase, we only included parents with a youngest child who was due to receive a vaccine in the next 6 months according to the Australian National Immunisation Program schedule (i.e. those aged either <18 months or between 3.5 and 4 y).^28^ To be eligible, parents also needed to provide consent for access to their youngest child’s AIR record. Due to the more limited pool of eligible panel members, no further quotas were placed on sampling. Exclusion criteria were parents aged <18 y, those where any member of their household had participated in phase two. Consent was implied via survey completion.

The phase three survey included a series of demographic questions, the VBAT draft 2 survey items, and the 15 items of the validated PACV vaccine hesitancy scale. The PACV is widely used to assess vaccine hesitancy, although it does not include any access items and has been shown to be predictive of future vaccine uptake in a United States parent population.^29^ The PACV was scored as described by its authors, resulting in a composite score from 0 to 100 where ≥50 is considered hesitant.^29^ Parents also provided their postcode and child’s first name to enable access to their child’s AIR record to allow future vaccine uptake to be accurately assessed. This was classified as “vaccinated on time” (received all vaccines within 1 month of the vaccines being due); “delayed” (received vaccines >1 month after the due date); or “not vaccinated” (had not received due vaccines at the time of database review). Each child’s AIR record was reviewed at least 1 month after the last eligible vaccine was due. We calculated the correlation between individual VBAT items and on-time vaccination. Those who received the vaccine late were combined with those who did not receive the vaccine within the study period for the purposes of this calculation.

The investigator team undertook a modified Nominal Group Technique approach^30^ to reach consensus on a final short-form VBAT (fewer than 10 items) and long form VBAT (more than 10 items). First, we each independently drafted potential models for the short- and long-form VBAT, based on individual item correlation with uptake and reflecting key theoretical and practical concepts or domains.

We then used logistic regression analysis to test the predictive validity of these models, as well as the PACV for comparison. Logistic regression helped determine the performance of these pre-defined models, with reference to the pseudo R^2^ value of Nagelkerke.^31^ We considered a pseudo R^2^ value of ≥0.1 to be a reasonably strong predictive performance. According to Hensher, Rose & Green,^32^ these values would correspond to classical shared variance (R^2^) values greater than 0.3, i.e., approximately three times higher than the average effect size in consumer behavior studies.^33^

To validate the dimensional structure of the VBAT long form, we conducted a confirmatory composite analysis (CCA),^34^ a kind of structural equation modeling that enables empirical validation of both traditional latent variables, such as vaccine confidence, and formative constructs, like vaccine accessibility.^35,36^ Formative constructs are defined by their indicators (i.e., the indicators are not assumed to represent a defined latent variable, but rather are designed to constitute the variable). Because these indicators need not be inter-correlated, analytic methods based on inter-item associations, like factor analysis and item response models, may be uninformative regarding the validity and reliability of instruments that capture formative constructs. The justification for aggregating indicator scores for formative constructs into a composite is not that they uniformly represent an underlying construct, but because the aggregate has utility, as determined by correlations with relevant criteria (i.e. PACV hesitancy score and/or timely vaccine uptake, as described above). Like all structural equation modeling techniques, CCA quantifies the degree of model fit based on the discrepancy between the model-implied and the empirical variance-covariance matrix. Values of the standardized root mean square residual (SRMR) below 0.05 combined with values of the comparative fit index (CFI) above 0.90 are regarded as acceptable.^37^

After discussing the results for each model, members of the core team individually drafted revised models and again brought them together for discussion and analysis until expert consensus was reached on the final items. The team discussed and agreed on qualitative labels for the domains identified through CCA.

Analysis for phases two and three was performed using SPSS 28^38^ and MPLUS 7.3.^39^

## Results

A total of 865 parents participated in the study (Table 1). Phase 2b parents were a sub-set of those participating in Phase 2a.

**Table 1.** Participant characteristics at each phase.

	PHASE 1: Cognitive testing		PHASE 2a: Validation of draft 1 item set (*n* = 532)	PHASE 2b: Re-test^ (*n* = 263)	PHASE 3: Validation of draft 2 item set	
	Australia (*n* = 18)	New Zealand (*n* = 10)			Participants wit AIR data^†^ (*n* = 156)	Participants without AIR data^†^ (*n* = 149)
Age of youngest child						
0–5 months	0	0	43 (8%)	26 (10%)	56 (36%)	21 (14%)
6–11 months	4 (22%)	4 (40%)	41 (8%)	21 (8%)	47 (30%)	50 (34%)
12–17 months	5 (28%)	3 (30%)	47 (9%)	33 (13%)	40 (26%)	54 (36%)
18–41 months	9 (50%)	3 (30%)	207 (39%)	114 (43%)	0%)	0%)
42–47 months	0	0	47 (9%)	19 (7%)	13 (8%)	24 (16%)
48–72 months	0	0	147 (28%)	50 (19%)	0%)	0%)
Socio-economic status/income‡						
High (least disadvantaged/$150,000+)	2 (11%)	1 (10%)	280 (53%)	138 (52%)	46 (30%)	58 (39%)
Medium ($75,000–$149,000)	3 (17%)	2 (20%)	124 (23%)	59 (22%)	63 (40%)	58 (39%)
Low (most disadvantaged/less than $74,000)	7 (39%)	7 (70%)	128 (24%)	66 (25%)	47 (30%)	33 (22%)
Missing	6 (33%)	0	0	0	0	0
Vaccine hesitancy level by VCF						
High (major concerns/delay or refuse vaccines)	6 (33%)	4 (40%)	76 (14%)	32 (12%)	11 (7%)	14 (9%)
Medium (minor concerns)	5 (28%)	3 (30%)	143 (27%)	73 (28%)	34 (22%)	35 (24%)
Low (no concerns)	7 (39%)	3 (30%)	313 (59%)	158 (60%)	109 (70%)	99 (66%)
Missing	0	0	0	0	2 (1%)	1 (1%)
Vaccine coverage level of residential area						
High	0	0	204 (38%)	81 (31%)	72 (46%)	48 (32%)
Medium	0	0	123 (23%)	76 (29%)	76 (49%)	83 (56%)
Low	0	0	205 (39%)	106 (40%)	6 (4%)	18 (12%)
Missing/not collected	18 (100%)	10 (100%)	0	0	2 (1%)	0 (0%)
Highest educational qualification						
High school certificate	2 (11%)	5 (50%)	63 (12%)	23 (9%)	24 (15%)	24 (16%)
Trade certificate or diploma	5 (28%)	3 (30%)	123 (23%)	67 (26%)	59 (38%)	31 (21%)
Undergraduate degree‡‡	11 (61%)	2 (20%)	191 (36%)	102 (39%)	40 (26%)	53 (36%)
Postgraduate degree	n/a	n/a	148 (28%)	67 (26%)	30 (19%)	36 (24%)
None of the above	0 (0%)	0 (0%)	6 (1%)	3 (1%)	3 (2%)	4 (3%)
Missing	0 (0%)	0 (0%)	1 (0%)	1 (0%)	0 (0%)	1 (0%)

^^^Subset of Phase 2a participants. ^†^Reasons for unavailable AIR data described in Results. ^‡^For phase 1, family income level was self-reported by participants. For phases 2 and 3, SEIFA level was calculated based on postcode.^27 ‡‡^The top two education level options were combined for Phase 1 participants as “Degree or higher.”

### Phase one: development of the item set

The global overview of systematic reviews of parent-level barriers to childhood vaccination included 30 reviews describing 64 unique barriers.^7^ When mapped to the Theoretical Domains Framework, these barriers aligned with 10 of the 14 domains. Four additional theoretical barriers were added to address Optimism, Intentions, Goals, and Behavioural Regulation.^22^ The 1-d workshop resulted in an initial set of 80 items (see supplemental files). Likert scales proposed as potential response options included scales in both directions (i.e. strongly disagree to strongly agree and vice versa), scales with or without a separate “Not sure/prefer not to say” option, 5-point scales with or 4-point scales without a neutral in-line option (e.g. “Neither agree nor disagree”), and display of options as a list rather than a horizontal scale.

In round 1 of cognitive testing, the whole initial item set was tested with Australian parents (*n* = 14) (Table 1). Six parents (43%) were highly vaccine hesitant (VCF levels 3–5), three (21%) spoke a language other than English, and nine (64%) had a bachelor’s degree or higher. Based on these interviews, we rephrased some items. We reduced the initial item set from 80 to 56 items, removing items that were poorly understood and those where a clearly preferred alternative phrasing was already included. The preferred response option was a vertical 4-point list, with a separate “not sure/prefer not to answer” option.

A second round of cognitive testing was undertaken with 14 parents from both Australia (*n* = 4) and New Zealand (*n* = 10). Four parents from New Zealand were highly vaccine hesitant (29%) and four had a bachelor’s degree or higher (29%). All the New Zealand parents self-identified as Māori and Pacific ethnicity. Following round 2, the items were again refined, and the item set was further reduced to 45 items (see supplemental files). The New Zealand parents often wanted an option to select “slightly agree” or “slightly disagree,” so the 4-point scale was revised to strongly agree, slightly agree, slightly disagree, and strongly disagree, with a “don’t know/can’t say” option.

### Phase two: reliability and validity of the VBAT items

A total of 532 parents completed the online survey of the 45 VBAT draft 1 items, with a subset of 263 (49%) completing the re-test 2 weeks later (Table 1). Characteristics of those who completed the re-test were similar to those who did not.

Twenty-four items demonstrated potential issues in either correlation with the VCF item (*n* = 11), test–retest reliability (*n* = 3), ceiling effect (*n* = 4), high numbers of “Don’t know/can’t say” responses (*n* = 11) or a combination of these. All these items were discussed with the investigator team, and the expert consensus was to remove 10 items, prioritizing removing those with high rates of “Don’t know/can’t say” responses (Table 2). The final number of items posted in this phase was 35.

**Table 2.** Characteristics of the VBAT draft 1 items reviewed for inclusion.

	Criteria for considering exclusion					
Reviewed items	Ceiling effect (>70% strongly agree)	High rate of “Don’t know/can’t say” (>5%)	Poor correla-tion with VCF (<0.2)	Poor test/retest (ICC <0.3)	Kept or cut	Rationale
I believe children’s immune systems respond well to vaccines even when they are very young	52.20%	**9.20%**	0.353	0.501	Cut	High ‘missing’ data
I believe my child is unlikely to experience a serious side effect from a vaccine	39.60%	**9.40%**	0.251	0.372	Cut	High ‘missing’ data
I believe people who do not vaccinate their children should be penalised	31.00%	**9.40%**	0.282	0.546	Cut	High ‘missing’ data
I believe vaccines can cause allergic conditions, for example asthma or eczema	20.00%	**13.40%**	0.248	0.482	Cut	High ‘missing’ data
I believe vaccines do not cause autism	51.80%	**14.20%**	0.32	0.398	Cut	High ‘missing’ data
I can find care for my other children when I need to vaccinate my child	48.00%	**17.60%**	**0.134**	0.353	Cut	High ‘missing’ data
I get information about vaccines from the radio, TV or newspaper	23.10%	2.70%	**0.09**	0.571	Cut	Cut because it does not provide meaningful actionable information for policymakers
I have a good relationship with my child’s doctor or nurse	59.80%	4.20%	**0.153**	0.384	Cut	Cut due to similarity to other items (e.g. “comfortable discussing vaccination”)
I prefer my child to get protection against many diseases through one combined vaccination rather than through separate vaccinations	40.80%	**12.60%**	**0.168**	0.428	Cut	High ‘missing’ data
My child has a severe fear of needles	21.80%	**6.40%**	**0.199**	0.594	Cut	High ‘missing’ data
I actively look for information about vaccines using social media	24.10%	2.90%	**0.099**	0.523	Kept	Not expected to be correlated to hesitancy so poor VCF correlation not relevant
I am comfortable discussing vaccination with my child’s doctor or nurse	**71.40%**	1.80%	0.276	**0.278**	Kept	Very close to threshold, retained
I believe children get too many vaccines in the first 2 y of life	22.00%	**5.50%**	0.326	0.543	Kept	Retained because previous research shows high association with vaccine hesitancy/refusal
I can afford any costs associated with vaccinating my child	52.40%	4.00%	**0.184**	0.538	Kept	Not expected to be correlated to hesitancy so poor VCF correlation not relevant
I can discuss vaccination in my preferred language with my child’s doctor or nurse	66.70%	**6.20%**	0.25	0.311	Kept	Important to retain for equity considerations (ie not relevant to majority, but important for minority groups)
I have enough knowledge to make a decision about vaccinating my child	58.20%	2.90%	0.224	**0.288**	Kept	Very close to threshold, retained
I know when my child’s vaccinations are due	56.90%	1.80%	**0.062**	0.442	Kept	Not expected to be correlated to hesitancy so poor VCF correlation not relevant
I know where to go to get my child vaccinated	**71.20%**	2.70%	0.231	0.368	Kept	Considered important to retain for equity considerations
I would feel guilty if I did not vaccinate my child and they got a vaccine	**70.20%**	2.00%	0.259	0.434	Kept	Very close to threshold, retained
I would reschedule my child’s vaccination appointment if they had a cold	57.80%	4.00%	**0.051**	0.373	Kept	Not expected to be correlated to hesitancy so poor VCF correlation not relevant
It is easy to remember when my child’s vaccinations are due	42.00%	2.50%	**0.047**	0.422	Kept	Not expected to be correlated to hesitancy so poor VCF correlation not relevant
It is my responsibility to make sure my child is vaccinated on time	**71.80%**	2.00%	0.281	0.387	Kept	Very close to threshold, retained
My child’s doctor or nurse recommends vaccination	68.00%	4.80%	0.224	**0.218**	Kept	Retained because previous research shows high association with vaccine uptake
My religious or cultural beliefs influence my vaccination decisions	19.80%	**8.00%**	**0.171**	0.508	Kept	Important to retain for equity considerations (ie not relevant to majority, but important for minority groups)

^Bold = results fall outside stated parameters for review.

### Phase three: predictive validity of VBAT items and model composition

A total of 305 parents completed the online survey consisting of the 35 VBAT draft 2 items with the intention of validating responses against AIR data to assess their ability to predict behavior. However, data from *n* = 149 parents could not be used for assessing the predictive validity because the child’s AIR record was unable to be accessed (*n* = 61); the child’s record was a duplicate (*n* = 2); the child was ineligible as AIR data indicated that vaccines had been given prior to the survey (*n* = 79); or the date of the next vaccination was beyond the timeline of the study (*n* = 7). This left 156 parents with survey data able to be linked to the child’s AIR data. Compared to the parents who provided AIR data, those for whom we could not access AIR data were less likely to live in areas with the highest rates of vaccination and had fewer children under 5 months old. They were similar in all other characteristics (Table 1). Of the 156 parents included in Phase 3, 70.5% (*n* = 110) children were vaccinated on time; 20.5% delayed (*n* = 32) or 8.9% not vaccinated (*n* = 14).

**Figure 2.**
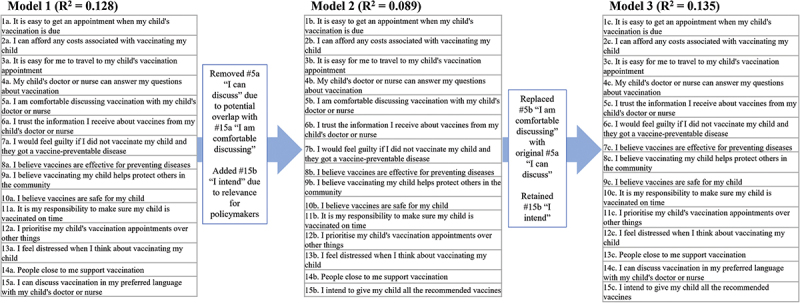
Example of model performance and refinement (long form).

Logistic regression analysis of the predictive performance of the team’s individual models for a VBAT long and short form informed refined models of each (see Figure 2 for example of the process). For benchmarking purposes, we calculated the PACV instrument’s pseudo R^2^ of Nagelkerke using data from our sample. The PACV generated a pseudo R^2^ value of 0.088.

**Figure 3.**
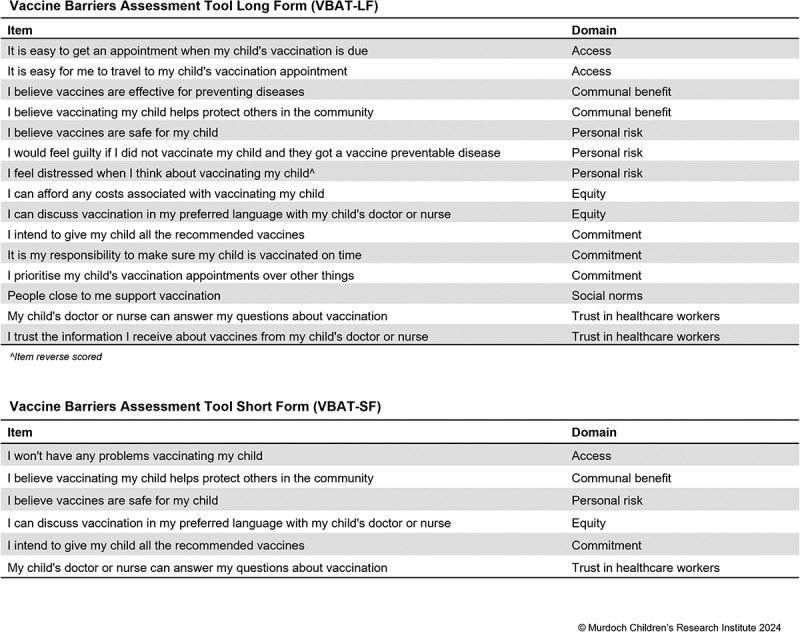
Short and long forms of the Vaccine Barriers Assessment Tool.

The team reached consensus on a final long- and short-form VBAT (Figure 3). The final short-form VBAT pseudo R^2^ value was 0.113, and the long form pseudo R^2^ was 0.135. The short VBAT includes six items representing six domains, while the long VBAT includes 15 items representing seven domains.

**Figure 4.**
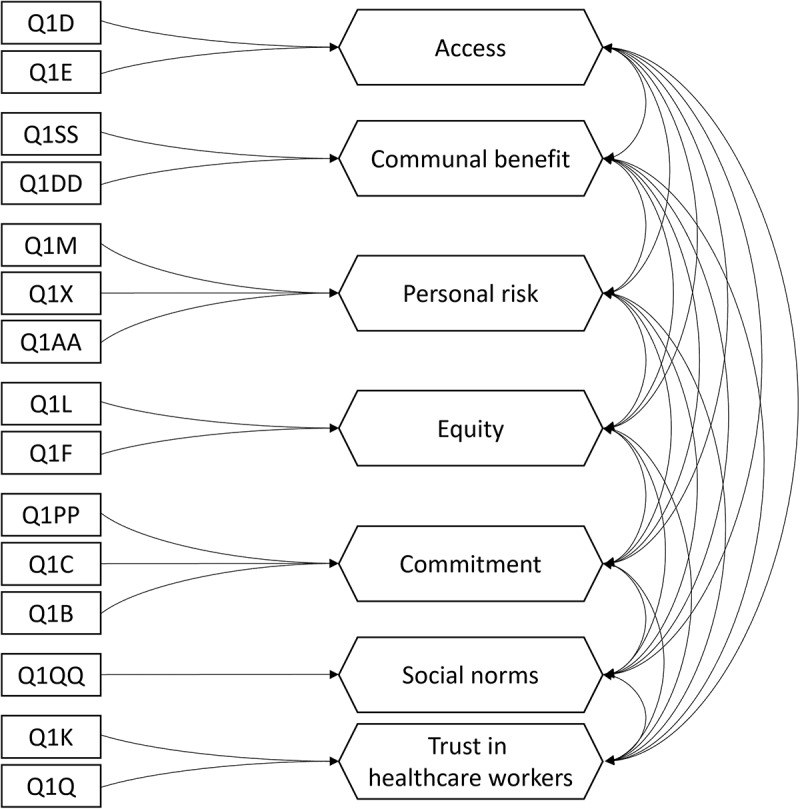
Confirmatory composite analysis of the Vaccine Barriers Assessment Tool (long form).

We validated the dimensional structure of the VBAT-LF (Figure 4) with data from all but one (*n* = 304) phase three participant (one participant had created too many missing values). Model estimation with maximum likelihood terminated normally and without any Heywood cases. While the test of exact fit was significant (χ^2^ = 188.878; df = 66; *p* < .001), a standardized root mean square residual of 0.045 and a comparative fit index of 0.928 indicated an acceptable model fit.

## Discussion

Understanding the reasons for under-vaccination is key to designing and targeting effective and cost-effective interventions. While vaccine hesitancy is often blamed for suboptimal coverage, the true barriers to uptake are often a complex mix of both access and acceptance issues. However, available measurement instruments focus almost exclusively on the psychosocial factors affecting uptake and do not include practical barriers to vaccination. We aimed to develop a measurement instrument to assess both access and acceptance barriers in the high-income country context of Australia, capitalizing on Australia’s unique national vaccine registry data for validation. Our robust and novel multi-phase process involved comprehensive review of theoretical and practical barriers to vaccination, cognitive item testing, and data-driven psychometric validation to produce a long 15-item form and a short 6-item form of the Vaccine Barriers Assessment Tool for Australia.

In our study population, both forms of the VBAT exhibited an acceptable level of predictive validity, both in absolute and in relative terms. In particular, both forms of the VBAT predicted vaccine uptake to a greater degree than the gold standard PACV tool, which measures vaccine hesitancy. The PACV is one of the most rigorously validated and widely used instruments in this field, with dozens of published studies reporting validation data of translated and adapted versions around the world.^40–44^ As with most vaccine hesitancy or confidence instruments, the PACV was developed through expert consultation and psychometric testing applying classical test theory methods. However, these methods can only be applied to psychological constructs like attitudes and beliefs. To incorporate access and equity barriers into our instrument, we had to consider different validation approaches. CCA is a novel form of structural equation modeling that has been designed for these kinds of problems. CCA is completely analogous to the well-known workhorse of psychometrics, confirmatory factor analysis, with the difference that CCA models composites instead of common factors. In the current setting, CCA helped to validate the VBAT and provide support for the VBAT’s dimensional structure. In addition to the novel application of CCA, we based our initial item set on both theoretical behavior change literature^22^ as well as published evidence on barriers to vaccination around the world.^7^ The aim of this approach was to capture the broadest possible set of barriers as an initial starting point, which we narrowed through several rounds of assessment with diverse parents, stratified by both socioeconomic and hesitancy factors.

Among the range of available measurement instruments, only the WHO BeSD quantitative data collection instrument also captures access barriers or practical issues. The VBAT domains align generally with the overarching BeSD categories, with some differences between the specific constructs and items. For example, the VBAT domains of “communal benefit” and “personal risk” are similar to BeSD constructs within the category of “what people think and feel.” Both instruments assess vaccine affordability, but this falls into the “equity” domain in VBAT and the “practical issues” category in the BeSD instrument. Developed by an expert global working group and cognitively tested in a range of countries, the BeSD tool is designed for flexible use primarily in low- and middle-income countries.^20,21^ The VBAT is not intended to replace the BeSD tool but is instead tailored specifically for the high-income country setting of Australia. The BeSD tool has been used in multiple studies since its launch in 2022 and formal validation is underway but has not yet been published. A unique feature of validation in Australia is the ability to use actual future vaccine uptake from the Australian Immunisation Register as the behavioral variable, as opposed to intention or self-reported vaccine uptake. Few countries have such a comprehensive register to support robust validation.

We created a short and a long form of the VBAT to maximize the ways in which it can be applied, following the examples of the BeSD and PACV instruments. The WHO BeSD working group note that countries may choose to use some items and not others as their setting requires but suggest including a minimum of five key indicators, while the PACV has 5-item and 15-item versions. The 16-item VBAT-LF includes a broader range of items, allowing for more specific identification of barriers. This specificity can improve cost-effective targeting of interventions. It can also identify areas, topics or populations where additional in-depth qualitative research may be needed. The VBAT-LF may be useful for researchers conducting primary research and intervention evaluations, where asking parents to spend 5–10 min on a survey is feasible. The breadth of factors assessed in the VBAT-LF makes it a potentially suitable evaluation tool for multicomponent interventions that involve both vaccine communication and service delivery elements, which may have different mechanisms of effect.^45^ We considered three factors when determining the length of the VBAT-SF: brevity/feasibility for use in population surveys; explanation of a high degree of variance (i.e., performance); and breadth of barriers assessed. Through discussion with the core study team and project advisory group, we determined that six items were the maximum length that would be feasible for most government surveys. We selected the six items that performed best and included both access- and acceptance-related items, which is the key point of differentiation from other scales. Since the domain of access contains more varied barriers than the other domains, we determined that a general accessibility item was more appropriate than selecting one type of access to assess in the VBAT-SF. Adding a social norms item lengthened the scale but did not increase its explanatory power. Where social norms are considered particularly relevant, we advise users to apply the VBAT-LF. Both forms include an intention item which can be used as a proxy for vaccine uptake if register data are not available.

Validated measurement instruments are increasingly relevant in a global era of data for decision-making and learning health systems. The COVID-19 pandemic highlighted policymakers’ and program managers’ need for surveillance of vaccine acceptance and accessibility issues to inform tailored and effective interventions. The VBAT is ideal for regular application to identify trends and changes in the vaccine uptake landscape, both at a national and regional level. The Australian Commonwealth has commissioned a pilot of this approach commencing in 2024, with an accompanying qualitative study to explore barriers in an identified priority population, e.g., certain cultural groups, and the VBAT-SF was applied in the 2023 New South Wales population health survey. Additional validation with a larger sample of Australian parents is also underway to inform the development of a scoring system for the VBAT. In addition to applying the current VBAT, several projects are underway to expand and adapt it for other populations, where the existing tool may not adequately capture the most relevant data. Validation in New Zealand is ongoing, with a specific focus on the Māori and Pacific Islander populations. In Australia, we are working with Aboriginal and Torres Strait Islander researchers and communities to create a culturally tailored version appropriate for this population, which may include alternative modes of administration (e.g. through individual or group discussions). We also aim to test the validity of the existing VBAT for childhood influenza vaccines and HPV vaccines, revising as necessary to capture unique barriers such as concerns about fertility or challenges related to the school-based consent process.

### Strengths and limitations

This study applied robust methods to develop and validate the VBAT, underpinned by expert opinion and consultation. However, there were some limitations. Many phases of the study were conducted during the COVID-19 pandemic. This may have had an impact on recruitment and parents’ willingness to consent to providing their child’s AIR data. It may also have affected some people’s vaccine perceptions or their vaccine uptake, though the vaccine uptake data were collected in 2022 when public health restrictions were no longer in place. We used an online panel to recruit parents, which allowed us to stipulate quotas for key sociodemographic characteristics. However, we were unable to recruit a large sample of parents of children with vaccines due in the next 6 months for Phase 3, due to the narrowness of this requirement. Predictive performance of individual items and models could change with larger or more diverse samples, and this is being explored with a larger study in 2024. We did not repeat the Phase 2 assessment with the whole sample from Phase 2a, but the characteristics between the two samples remained similar. In Phase 3, we defined people who did not receive a vaccine in the study period as ‘non-vaccinators,’ but it is possible that they may have vaccinated later and may therefore be ‘delayers’ instead. There could be relevant differences in how non-vaccinators and delayers answer the VBAT items, which this study could not capture or consider. We addressed the unique challenges of validating a tool that combines both psychological constructs and practical issues by utilizing novel techniques drawn from other fields of research, but we were unable to apply classical test theory, so our validation process had to be slightly adapted so that it was also applicable to formative constructs. Finally, we have only validated the survey in Australia and in English. Additional validation should be undertaken with any translations or application in other countries.

## Conclusion

The VBAT is the first instrument to measure both access and acceptance barriers to childhood vaccination that has been developed and validated in a high-income country setting against vaccine behavior using immunization registry data. It will be a valuable tool for serial surveillance at a national and regional level to inform vaccine policy and programs and as a measure for evaluating interventions. Future adaptations for other populations, such as Indigenous parents, and additional validation in Australia and New Zealand are underway.

## Supplementary Material

Supplemental documents.docx

## Data Availability

The data that support the findings of this study are available from the corresponding author, JK, upon reasonable request.
