# Supplementary material for: Development and validation of the Vaccine Barriers Assessment Tool for identifying drivers of under-vaccination in children under five years in Australia
Source: Hum Vaccin Immunother. 2024 Jun 6;20(1):2359623. doi: 10.1080/21645515.2024.2359623 (PMC11164230; doi:10.1080/21645515.2024.2359623)
Supplement: Supplemental documents.docx [file KHVI_A_2359623_SM3413.docx]

# **Supplemental document 1 – items carried through development process**

| **PHASE 1 INITIAL ITEMS TAKEN TO COGNITIVE TESTING (n=80)** | **CARRIED TO ROUND 2 COGNITIVE TESTING (n=56)** | **CARRIED TO PHASE 2 (n=45)** | **CARRIED TO PHASE 3 (n=35)** | **INCLUDED IN FINAL VBAT LF (n=15)** | **INCLUDED IN FINAL VBAT SF (n=6)** |
| --- | --- | --- | --- | --- | --- |
| It is easy to vaccinate my child | X | X | X | X | X |
| I won't have any problems vaccinating my child | ✓ | ✓ | ✓ | X | ✓ |
| I intend to vaccinate my child | REWORDED: I intend to give my child all the recommended vaccines | ✓  (reworded version) | ✓  (reworded version) | ✓  (reworded version) | ✓  (reworded version) |
| I plan to vaccinate my child on time | X | X | X | X | X |
| I have a plan for when to vaccinate my child | X | X | X | X | X |
| I have a plan for how to get my child vaccinated | X | X | X | X | X |
| Vaccinating my child on time is a high priority | X | X | X | X | X |
| I prioritise my child’s vaccination appointments over other things | ✓ | ✓ | ✓ | ✓ | X |
| It is easy to find the time (in my schedule) to vaccinate my child | X | X | X | X | X |
| Vaccinating my child is quick | X | X | X | X | X |
| I can find time to vaccinate my child | ✓ | X | X | X | X |
| It is easy for me to get an appointment to vaccinate my child | REWORDED: It is easy to get an appointment when my child's vaccination is due | ✓  (reworded version) | ✓  (reworded version) | ✓  (reworded version) | X |
| For me, getting an appointment to vaccinate my child is easy | X | X | X | X | X |
| I can vaccinate my child when it suits me | X | X | X | X | X |
| It is easy for me to travel to my child's vaccination appointment | ✓ | ✓ | ✓ | ✓ | X |
| I can vaccinate my child close to home | X | X | X | X | X |
| I don’t need to travel far to vaccinate my child | X | X | X | X | X |
| It is easy for me to pay for my child's vaccination appointment | X | X | X | X | X |
| Vaccinating my child is affordable | REWORDED: I can afford any costs associated with vaccinating my child | ✓  (reworded version) | ✓  (reworded version) | ✓  (reworded version) | X |
| I can easily pay for my child’s vaccination appointment | X | X | X | X | X |
| Finding childcare for my other children makes it difficult to vaccinate my child | X | X | X | X | X |
| It is easy to arrange childcare for my other children when taking my child to their vaccination appointment | X | X | X | X | X |
| I can find care for my other children when I need to vaccinate my child | ✓ | ✓ | X | X | X |
| My child's provider makes it easy for me to discuss vaccination with them | X | X | X | X | X |
| It is easy to discuss vaccination with my child’s doctor or immunisation nurse | REWORDED: I am comfortable discussing vaccination with my child's doctor or nurse | ✓  (reworded version) | ✓  (reworded version) | X | X |
| I have a good relationship with my child's provider [doctor or immunisation nurse] | REWORDED: I have a good relationship with my child's doctor or nurse | ✓  (reworded version) | X | X | X |
| My child's provider recommends vaccination | REWORDED: My child's doctor or nurse recommends vaccination | ✓  (reworded version) | ✓  (reworded version) | X | X |
| My child's provider can answer my questions about vaccination | REWORDED: My child's doctor or nurse can answer my questions about vaccination | ✓  (reworded version) | ✓  (reworded version) | ✓  (reworded version) | ✓  (reworded version) |
|  | ADDED VARIATION: My child's doctor or nurse answers my questions about vaccination | X | X | X | X |
| I can discuss vaccination in my preferred language at my child's clinic | X | X | X | X | X |
| I can discuss vaccination in my preferred language with my child’s doctor or immunisation nurse | ✓ | ✓ | ✓ | ✓ | ✓ |
| I believe vaccines are safe for my child | REWORDED: I believe vaccines are safe for most children | REWORDED #2 (same as original): I believe vaccines are safe for my child | ✓  (reworded #2 version) | ✓  (reworded #2 version) | ✓  (reworded #2 version) |
|  | ADDED VARIATION: I believe childhood vaccines are safe | X | X | X | X |
| It is better for my child to develop immunity by getting vaccinated than by getting sick | REWORDED: I prefer for my child to develop immunity by getting vaccinated rather than by getting sick from the disease | ✓ | ✓ | X | X |
| Children get better immunity from vaccines than through natural immunity from diseases | X | X | X | X | X |
| I believe vaccines strengthen my child's immune system | ✓ | ✓ | ✓ | X | X |
| I believe vaccines make my child's immune system weaker | X | X | X | X | X |
| I trust the science about vaccines | ✓ | ✓ | ✓ | X | X |
| I trust the information I receive about vaccines from my child's doctor or nurse | ✓ | ✓ | ✓ | ✓ | X |
| I trust the information I receive about vaccines from the government | X | X | X | X | X |
| I trust the information I receive about vaccines from pharmaceutical companies | X | X | X | X | X |
| I am comfortable with the number of vaccines my child receives | REWORDED: I believe children get too many vaccines in the first two years of life | ✓ | ✓ | X | X |
| I am comfortable with the age at which my child receives vaccines | REWORDED: I believe children’s immune systems are strong enough to have vaccines when they’re very young | REWORDED #2: I believe children's immune systems respond well to vaccines even when they are very young | X | X | X |
| It is better for my child to receive one combined vaccine than several separate injections | REWORDED: I prefer my child to receive one combined vaccine than several separate injections | REWORDED #2: I prefer my child to get protection against many diseases through one combined vaccination rather than through separate vaccinations | X | X | X |
| I feel equipped to make good decisions about my child’s vaccines | REWORDED: I feel confident to make a decision about my child’s vaccinations | ✓  (reworded version) | ✓  (reworded version) | X | X |
| The pain of vaccine needles is not too much for my child to handle | REWORDED: The pain of vaccine needles is too much for children to handle | ✓  (reworded version) | ✓  (reworded version) | X | X |
| My child can handle the pain of vaccine needles | X | X | X | X | X |
| The schedule of recommended vaccines is appropriate for my child | ✓ | ✓ | ✓ | X | X |
| I feel distressed when I think about vaccinating my child | ✓ | ✓ | ✓ | ✓ | X |
| I believe that vaccines do not cause autism | ✓ | ✓ | ✓ | X | X |
| I support government policies that require vaccination | X | X | X | X | X |
| I believe people who do not vaccinate their children should be penalised | ✓ | ✓ | X | X | X |
| If I did not vaccinate my child, I would feel very bad if they caught a vaccine-preventable disease | REWORDED: I would feel guilty if I did not vaccinate my child and they got a vaccine-preventable disease | ✓  (reworded version) | ✓  (reworded version) | ✓  (reworded version) | X |
| I would feel very bad if my child had a reaction to a vaccine | REWORDED: I would feel guilty if my child had a bad reaction to a vaccine | X | X | X | X |
| I would reschedule my child's vaccination appointment if they had a cold | ✓ | ✓ | ✓ | X | X |
| I believe the diseases prevented by vaccines are serious | ✓ | X | X | X | X |
| My child could get a serious disease if they are not vaccinated | ✓ | ✓ | ✓ | X | X |
| Vaccines are effective for preventing diseases | REWORDED: I believe vaccines are effective for preventing diseases | ✓  (reworded version) | ✓  (reworded version) | ✓  (reworded version) | X |
| I do not believe that vaccines cause allergies | REWORDED: I believe vaccines can cause allergies | REWORDED #2: I believe vaccines can cause allergic conditions, for example asthma or eczema | X | X | X |
| My child is unlikely to experience a serious side effect from a vaccine | REWORDED: I believe my child is unlikely to experience a serious side effect from a vaccine | ✓  (reworded version) | X | X | X |
| I have a needle phobia | X | X | X | X | X |
| My child has a needle phobia | REWORDED: My child has a severe fear of needles | ✓  (reworded version) | X | X | X |
| I have enough knowledge to make a decision about vaccinating my child | ✓ | ✓ | ✓ | X | X |
| It is easy to remember to make vaccination appointments for my child | REWORDED: It is easy to remember when my child’s vaccinations are due | ✓  (reworded version) | ✓  (reworded version) | X | X |
| It is easy to remember to attend vaccination appointments for my child | X | X | X | X | X |
| I know where to find accurate information about vaccination, if I need it | REWORDED: I know where to find trustworthy information on vaccines | ✓  (reworded version) | ✓  (reworded version) | X | X |
| I have access to enough information to make a decision about vaccinating my child | X | X | X | X | X |
| I know when my child's vaccinations are due | ✓ | ✓ | ✓ | X | X |
| I am satisfied with the information I have received about vaccination | REWORDED: I am satisfied with the information I have received from my doctor or nurse about vaccination | ✓  (reworded version) | ✓  (reworded version) | X | X |
|  | ADDED VARIATION: I am satisfied with the information I have accessed about vaccination | X | X | X | X |
|  | ADDED VARIATION: I am satisfied with the information I have found or received about vaccination | X | X | X | X |
| I know where to go to get my child vaccinated | ✓ | ✓ | ✓ | X | X |
| The information I have received about vaccinations from my child's doctor or immunisation nurse is reliable | X | X | X | X | X |
| I get information about vaccines from social media | REWORDED: I actively look for information about vaccines using social media | ✓  (reworded version) | ✓  (reworded version) | X | X |
| I get information about vaccines from the radio, TV or newspaper | ✓ | ✓ | X | X | X |
| It is my responsibility to make sure my child is vaccinated on time | ✓ | ✓ | ✓ | ✓ | X |
| I do my own research about vaccination | X | X | X | X | X |
| My family thinks I should vaccinate my child | REWORDED: Most of my friends and family support vaccination | REWORDED #2: People close to me support vaccination | ✓  (reworded #2 version) | ✓  (reworded #2 version) | X |
| People close to me think vaccination is a good idea | X | X | X | X | X |
| People in my community support vaccination | X | X | X | X | X |
| My religious beliefs support vaccination | REWORDED: My religious or cultural beliefs support vaccination | X | X | X | X |
| Leaders in my religion support vaccination | REWORDED: My religious or cultural beliefs influence my vaccination decisions | ✓  (reworded version) | ✓  (reworded version) | X | X |
| It is important for my child to be vaccinated to protect others in the community | REWORDED: I believe vaccinating my child helps protect others in the community | ✓  (reworded version) | ✓  (reworded version) | ✓  (reworded version) | ✓  (reworded version) |
| Because other children are vaccinated, it is not necessary to have my child vaccinated | REWORDED: My child does not need to be vaccinated if most children are vaccinated | X | X | X | X |
